# Molecularly targeted protease-activated probes for visualization of glioblastoma: a comparison with 5-ALA

**DOI:** 10.3171/2024.1.JNS231137

**Published:** 2024-03-29

**Authors:** Dora Konečná, Petr Výmola, Nikola Ternerová, Barbora Výmolová, Elena Garcia-Borja, Rosana Mateu, Filip Šroubek, Jan Pankrác, John C. Widen, Matthew Bogyo, David Netuka, Petr Bušek, Aleksi Šedo

**Affiliations:** 1 Department of Neurosurgery and Neurooncology, First Faculty of Medicine and Military University Hospital, Prague;; 2 Institute of Biochemistry and Experimental Oncology, First Faculty of Medicine, Charles University, Prague;; 3 Institute of Information Theory and Automation, Czech Academy of Sciences, Prague;; 4 Center of Advanced Preclinical Imaging, First Faculty of Medicine, Charles University, Prague, Czech Republic; and; 5 Department of Pathology, Stanford University School of Medicine, Stanford, California

**Keywords:** fluorescence-guided surgery, glioblastoma, 5-ALA, mouse models, activity-based probes, cathepsin, oncology, 5-ALA = 5-aminolevulinic acid, EGFR = epidermal growth factor receptor, em = emission, ex = excitation, FGS = fluorescence-guided surgery, GBM = glioblastoma, ICG = indocyanine green, iMRI = intraoperative MRI, iUSG = intraoperative ultrasonography, NIR = near-infrared, PPIX = protoporphyrin IX, PRE = pharmacoresistant epilepsy, TNR = tumor to normal brain tissue ratio

## Abstract

**OBJECTIVE:**

The highly infiltrative growth of glioblastoma (GBM) makes distinction between the tumor and normal brain tissue challenging. Therefore, fluorescence-guided surgery is often used to improve visual identification of radiological tumor margins. The aim of this study was to evaluate the ability of recently developed molecularly targeted near-infrared (NIR) protease-activated probes to visualize GBM tissue and to compare the most promising candidate with the gold standard, 5-aminolevulinic acid (5-ALA).

**METHODS:**

Single-substrate probes 6QC-ICG and 6QC-Cy5 (cysteine cathepsin cleavable), double-substrate probes AG2-FNIR and AG2-Cy5 (cysteine cathepsin and caspase 3 cleavable), and 5-ALA were administered intravenously to mice with orthotopic tumors. Activation of the probes was also evaluated in cell cultures in vitro and in biopsy material from patients with GBM ex vivo. The tumor to normal brain tissue fluorescence ratio (TNR) was quantified in brain sections using preclinical and clinical visualization platforms, and in tissue homogenates and cell suspensions using spectrofluorimetry. Subcellular localization of the fluorophores was visualized by confocal microscopy.

**RESULTS:**

In vitro, the single-substrate probe 6QC-ICG was cleaved in glioma cells and macrophages, and the resulting fluorophore accumulated intracellularly. In experimental GBMs, both single- and double-substrate probes visualized tumor tissue, while in healthy brain tissue the signal was minimal. TNR was highest for 6QC-ICG and AG2-FNIR, but the signal intensity was higher for 6QC-ICG. Using xenograft and syngeneic mouse models, as well as human GBM biopsy material ex vivo, the authors confirmed the ability of 6QC-ICG to specifically visualize the glioma tissue using preclinical and clinical visualization platforms. Finally, a comparison with 5-ALA in animals coadministered with both compounds revealed a higher TNR for 6QC-ICG in experimental GBMs.

**CONCLUSIONS:**

The cysteine cathepsin–cleavable probe 6QC-ICG is activated by glioma cells and tumor-associated macrophages, leading to a high contrast between tumor and nontumorous brain tissue that is superior to that of the current standard, 5-ALA. In addition to a well-defined mechanism of action, protease-activated probes that use NIR fluorophores (e.g., indocyanine green) have the advantage of low absorption and scattering of the NIR light and lower tissue autofluorescence. These results suggest that 6QC-ICG has the potential to become the targeted agent in intraoperative detection of GBM tissue using fluorescence imaging.

## In Brief

This study evaluated the efficacy of several near-infrared protease-activated probes for intraoperative visualization of glioblastoma (GBM) and compared the most promising candidate with the gold standard, 5-aminolevulinic acid (5-ALA). The cysteine cathepsin-cleavable probe 6QC-ICG was activated in experimental GBMs and human GBM tissue ex vivo and provided higher contrast between tumor and healthy tissue than 5-ALA. These findings indicate the potential of 6QC-ICG to become a targeted agent for fluorescence-guided surgery in GBM.

Surgery remains the mainstay of treatment for glioblastoma (GBM), the most malignant and most common primary brain tumor in adults. Although patient prognosis is poor, surgery combined with radiotherapy and chemotherapy prolongs survival and improves quality of life.^1^ GBMs are highly invasive tumors with glioma cells spread up to approximately 2 cm beyond the gadolinium-enhancing tumor.^2^ For GBMs distant from eloquent areas, a supratotal resection of the surrounding tissue is feasible.^3–5^ However, for tumors located close to eloquent areas, this approach is not possible because of postoperative complications, such as neurological deficits. The main goal of GBM surgery is gross-total resection, i.e., maximum safe removal of the gadolinium-enhancing tumor.^6^ Intraoperative differentiation between GBM and normal brain tissue is challenging. Several methods have therefore been developed to help neurosurgeons achieve maximal safe resection. These include neuronavigation,^7^ intraoperative MRI (iMRI),^8^ intraoperative ultrasonography (iUSG),^9^ and fluorescence-guided surgery (FGS).^10,11^ Notably, FGS offers the advantage of enabling real-time visualization of tumor tissue during surgery.

Currently, the only contrast agent widely used for FGS in GBMs is 5-aminolevulinic acid (5-ALA). 5-ALA accumulates in highly metabolically active tissues and is converted to protoporphyrin IX (PPIX).^12^ After excitation with blue light (λ = 410–420 nm), PPIX emits light in the red spectrum (λ = 635 nm). The use of 5-ALA helps the surgeon distinguish GBM from normal brain tissue, increases the likelihood of achieving gross-total resection, and leads to a prolonged progression-free survival.^13^ However, the limitations of 5-ALA must also be considered. Because of diffusion, the PPIX signal usually extends beyond the gadolinium-enhancing tumor,^10^ which may lead to the removal of eloquent structures. While highly vascularized tumor tissue exhibits strong PPIX fluorescence,^10^ the transition zone containing infiltrating tumor cells and edematous brain tissue shows only a diffuse pink staining. This, combined with the stronger autofluorescence of nontumorous tissue in the blue light, reduces the specificity of 5-ALA.^14,15^ Furthermore, the contrast is highest after 2–8 hours and diminishes significantly with time.^16^ Therefore, if surgery is delayed beyond 12 hours, it should be postponed, with 5-ALA redosing after at least 24 hours.

Other agents, such as fluorescein and high-dose indocyanine green (ICG), have been tested for intraoperative visualization of gliomas.^17,18^ ICG accumulates in the gadolinium-enhancing tumor tissue, but the precise mechanism is not fully understood.^17,18^

The relatively nonspecific mechanism of action of current imaging agents has prompted the development of compounds targeting specific molecules overexpressed in the GBM microenvironment. While various cell surface proteins, such as the epidermal growth factor receptor (EGFR)^19,20^ or integrins,^21^ typically bind only in a 1:1 ratio to the corresponding imaging probes, proteases have the potential to produce signal amplification through their enzymatic activity. In substrate-based probes containing a fluorophore linked through a short peptide to a quencher, proteolytic removal of the quencher activates the probe in the tumor, whereas in the surrounding healthy tissue the probe remains unprocessed, and thus optically silent.^22^

The recently developed substrate-based probe 6QC-ICG contains the FDA-approved near-infrared (NIR) fluorophore ICG and has been shown to be activated by cysteine cathepsins in tumor tissue.^23^ In addition to probes activated by one proteolytic cleavage such as 6QC-ICG (single-substrate probes), probes requiring proteolytic processing by two orthogonal proteases (double-substrate probes, AND-gate) have recently been developed.^22,24^ The proof-of-principle double-substrate probes requiring cleavage by cathepsins and caspase 3 have shown promise in enhancing the contrast between tumor and healthy tissue in breast and lung cancer models.^22^ So far, there have been no studies exploring the use of these probes in GBM, which exhibits increased levels of cysteine cathepsins,^25^ but differs from extracranial malignancies in several aspects.

The aim of our study was to evaluate the ability of recently developed single- and double-substrate probes to be specifically activated in brain tumor tissue and to compare the most promising candidate with the most commonly used agent, 5-ALA.

## Methods

See the Supplementary Methods for a detailed account of the methods used in this study.

### Tissue Samples

Biopsy material was obtained in accordance with the Declaration of Helsinki from consenting patients with newly diagnosed GBM (2021 WHO classification^26^) or pharmacoresistant epilepsy (PRE) in the Department of Neurosurgery and Neurooncology, Military University Hospital, Prague.

### Cell Culture

U251 (LGC Standards) and Gl261 (Division of Cancer Treatment and Diagnosis Tumor Repository, National Cancer Institute) glioma cells were cultured under standard conditions. Glioma stem-like cell line NCH644 (Cell Lines Service) and glioma stem-like cells B-6 derived from human GBM were cultured as previously described.^27^

M2-polarized macrophages were differentiated from monocytes from buffy coats of consenting healthy donors (Institute of Hematology and Blood Transfusion, Prague).

### Animals and Ethics Statement

All animal experiments were performed in accordance with protocols approved by the Charles University Commission at the First Faculty of Medicine and the Ministry of Education, Youth and Sports of the Czech Republic. Surgical procedures were performed under anesthesia (ketamine 100 mg/kg and xylazine 10 mg/kg) in immunodeficient [NOD.129S7(B6)-Rag1tm1Mom/J] and immunocompetent (C57BL/6J) mice.

### Orthotopic GBM Models

Glioma cells in serum-free media were stereotactically injected into mouse brains (right hemisphere, 3 mm caudal to bregma, 2.5 mm lateral to the sagittal suture, 3 mm deep).^28^ Experiments with fluorescent agents were performed when symptoms appeared.

### Administration of the Fluorescent Agents

Protease-activated probes 6QC-ICG, 6QC-Cy5, AG2-FNIR, AG2-Cy5 (10–30 nmol per mouse),^23^ and 5-ALA (Gliolan 30 mg/ml, 1 × 1.5 g, Photonamic GmbH; 5-ALA 100 mg/kg^29^) were administered via the tail vein. For experiments comparing 6QC-ICG with 5-ALA, both substances were sequentially coadministered to tumor-bearing animals 6 and 5 hours prior to euthanasia, respectively.

### Fluorescence Imaging

Extracted brains or coronal plane cryosections (Cryotome Leica CM1950) were visualized using Xtreme (Bruker BioSpin; excitation/emission [ex/em] 630/700 nm for Cy5, ex/em 730/790 nm for ICG and FNIR) or ChemiDoc MP (Bio-Rad; ex/em 755–777/813–860 nm). Tumor location was validated in the same cryosections after staining with a nuclear dye (TO-PRO-3 Iodide, Thermo Fisher Scientific). To evaluate the signal at the tumor margins, a mask highlighting the tumor was generated in the nuclear dye–stained images and transferred into the corresponding position in the ICG images.

To visualize 6QC-ICG on clinical-grade platforms, we used coronal sections of tumor-bearing brains and the DaVinci Xi system (Intuitive Surgical, Inc.; maximum ex/em ~800 nm/600–900 nm) and the NIR mode (ex/em 730–740 nm/>800 nm) on the ORBEYE exoscope (Olympus). To visualize 5-ALA, we used a blue-light mode (ex/em 410–420/>450 nm) of the ORBEYE exoscope.

### Quantitative Image Analysis

Images were analyzed using ImageJ (National Institutes of Health). The tumor and contralateral hemisphere (normal brain tissue) were used to calculate the tumor to normal brain tissue ratio (TNR). To compare 6QC-ICG and 5-ALA, we used images captured from identical positions in the visible light, NIR, and blue-light modes of the ORBEYE exoscope. For 6QC-ICG, TNR was calculated as described above. For 5-ALA, RGB (red-blue-green) images were converted to grayscale by calculating the scalar product of the pixel colors and a projection RGB vector (defined as the vector that best separates colors in the tumor region from colors in a healthy brain tissue region). For each sample, we computed a single-projection vector by solving an optimization problem, which maximized the distance between the median colors of both regions. The mean projection vector was then used to perform the grayscale conversion of all RGB images.

### Immunohistochemistry

Human cells were detected using the anti–human nuclei antibody (MAB4383, Sigma-Aldrich).^30^

### 6QC-ICG Activation in Ex Vivo Human GBM Tissue

Pieces of freshly resected GBM or PRE were incubated with 1 µM 6QC-ICG at 37°C and imaged using ChemiDoc MP (ex/em 755–777/813–860 nm). The mean fluorescence intensity was calculated using the Image Lab software (Bio-Rad). Because simultaneous comparison for individual human tissues was not logistically feasible, we used the mean fluorescence intensity measured in healthy mouse brain tissue processed with the human material to normalize fluorescence intensities in individual experiments.

### 6QC-ICG Activation In Vitro

Cells were incubated with 1 µM 6QC-ICG for 6 hours. Fluorescence was measured in cell suspensions using the Infinite M1000 (Tecan) at ex/em 795/823 nm or analyzed by flow cytometry (BD FACSVerse, BD Biosciences; 640-nm laser, APC-Cy7).

### Confocal Microscopy

Cells were imaged after 2 hours of incubation with 1 µM 6QC-Cy5 using a FluoView FV300 confocal microscope (Olympus; 633-nm laser).

### Spectrofluorimetric Analysis of Tissue Homogenates

Experimental tumors and healthy mouse brain tissue were mechanically homogenized, and fluorescence was measured using the Infinite M1000 (ex/em 795/823 nm for 6QC-ICG, ex/em 402/635 nm for 5-ALA). Data were normalized to the weight of the tissue sample. Normalization to total protein produced similar results.

### Statistical Analysis

Statistical analyses were performed with Statistica 14 (Tibco Software) and GraphPad Prism 8.0.2 (GraphPad Software). A two-sided p value < 0.05 was considered statistically significant.

## Results

### Glioma Cells and M2-Polarized Macrophages Activate 6QC-ICG

**FIG. 1.**
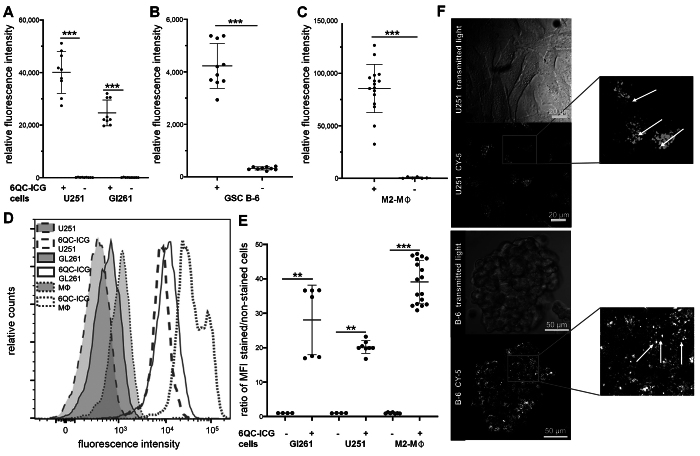
6QC-ICG is activated in glioma cells and tumor-associated macrophages. **A–C:** Fluorescence intensity in U251 and Gl261 glioma cell lines (A), glioma stem-like cells B-6 (B), and primary M2-polarized macrophages (M2-MΦ) (C) incubated with or without 1 µM 6QC-ICG for 6 hours. Fluorescence was measured in cell suspensions using spectrofluorimetry at ex/em 795/823 nm. Data are presented as mean ± SD from three independent experiments performed in triplicate. The *horizontal lines* represent the mean, the *error bars* the SD, and the *dots* the raw data. ***p < 0.001, Mann-Whitney U-test. **D:** Flow cytometry analysis (ex/em 640/783) of U251 and Gl261 glioma cell lines and primary M2-polarized macrophages incubated with or without 1 µM 6QC-ICG for 6 hours. **E:** The mean fluorescence intensity (MFI) ratio of stained to unstained cells was used to quantify the increase in fluorescence in individual cell lines; data are from two independent experiments performed at least in triplicate. ***p < 0.001, **p < 0.01, Mann-Whitney U-test. **F:** Representative images of U251 cells and a glioma stem-like cell B-6 spheroid incubated with 1 µM 6QC-Cy5 for 2 hours. *White arrows* show the fluorescence signal in granular vesicle-like structures.

We evaluated the ability of a recently described single-substrate probe 6QC-ICG (cysteine cathepsin cleavable) to be activated by cells present in the GBM microenvironment. We incubated glioma cells, glioma stem-like cells, and primary M2-polarized macrophages with 6QC-ICG, and measured fluorescence using spectrofluorimetry. Autofluorescence of the cells was minimal, and we found a large increase of fluorescence in all cell types incubated with 6QC-ICG (Fig. 1A–C).

To further validate activation of the probe in glioma cells and macrophages, we used flow cytometry. Consistent with the spectrofluorimetry data, we observed an increase of fluorescence in all evaluated cell types incubated with 6QC-ICG. The most pronounced increase was observed in M2-polarized macrophages (Fig. 1D and E).

The fluorescent products of 6QC-based probes accumulate in the lysosomes because of their positive charge (latent lysosomotrophic effect^31^), which results in an improved intracellular retention. To verify this in glioma cells, we incubated U251 and glioma stem-like cells B-6 with 6QC-Cy5, which has spectral characteristics better suited to confocal microscopy imaging. In line with our expectations, we observed that the fluorescence signal was concentrated in vesicle-like structures within glioma cells (Fig. 1F). These data indicate that both glioma cells and tumor-associated macrophages can activate 6QC-ICG.

### Protease-Activated Substrate Probes Visualize Tumor Tissue in Orthotopic Mouse GBM Models

We further assessed the ability of single-substrate (6QC-ICG and 6QC-Cy5) and double-substrate (AG2-FNIR and AG2-Cy5)^22^ probes to visualize tumor tissue using a syngeneic Gl261 GBM model.^32^ Tumor-bearing mice were euthanized, and extracted brains were visualized 2 and 24 hours after intravenous administration of the compounds.

**FIG. 2.**
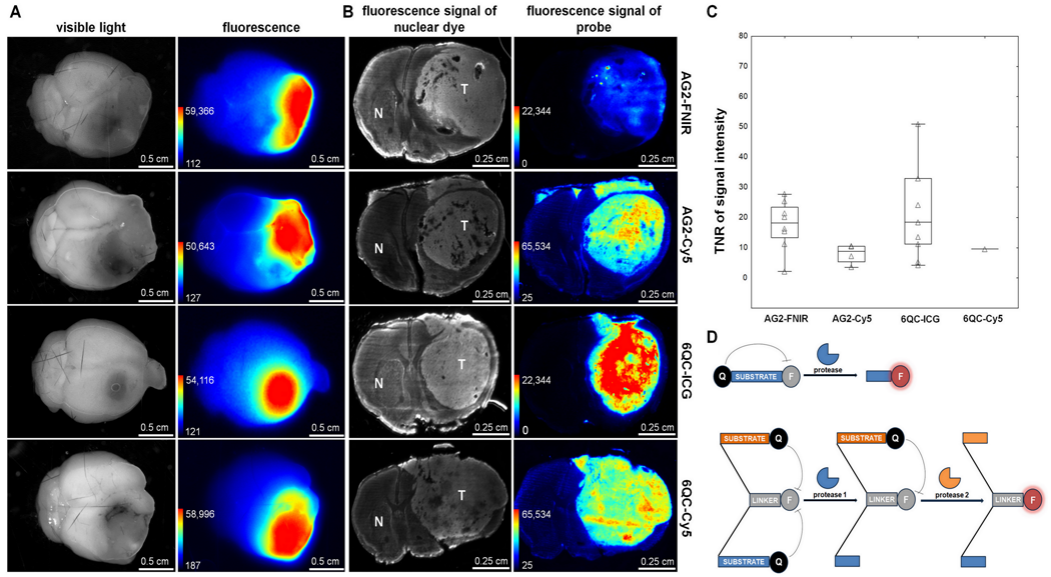
Activation of protease-activated substrate probes in an orthotopic GBM model. **A:** Images of brains with Gl261 tumors visualized using visible light (*first column*) and fluorescence (*second column*). The Xtreme imaging system was used to visualize fluorescence at ex/em 730/790 nm (AG2-FNIR and 6QC-ICG) and ex/em 630/700 nm (AG2-Cy5 and 6QC-Cy5). **B:** Fluorescence signals of the nuclear dye showing the location of Gl261 tumors in brain cryosections (*first column*) and the probes in corresponding brain cryosections (*second column*). **C:** Quantification of TNR for individual probes. The *horizontal lines* represent the median, the *boxes* the 25th to 75th percentile, the *whiskers* the nonoutlier range, and the *triangles* the raw data. Tumor-bearing mice were euthanized 24 hours after intravenous administration of 20 nmol of double-substrate probes AG2-FNIR and AG2-Cy5 (cysteine cathepsin and caspase 3 cleavable, AND-gate) and single-substrate probes 6QC-ICG and 6QC-Cy5 (cysteine cathepsin cleavable). The numbers of mice were as follows: 8 for AG2-FNIR, 9 for 6QC-ICG, 4 for AG2-Cy5, and 1 for 6QC-Cy5. Brains were extracted and 50-µm cryosections were prepared and imaged using ChemiDoc MP at ex/em 755–777/813–860 nm (AG2-FNIR and 6QC-ICG) and ex/em 625–650/675–725 nm (AG2-Cy5 and 6QC-Cy5). **D:** Schematic representation of single- and double-substrate probes. F = fluorophore; N = normal brain tissue; Q = quencher; T = tumor.

Fluorescence was clearly detectable in tumors in all animals at both time points and was stronger after 24 hours (data not shown), suggesting that the compounds are activated within the GBM microenvironment. Normal brain tissue exhibited minimal fluorescence for both single- and double-substrate probes (Fig. 2).

**FIG. 3.**
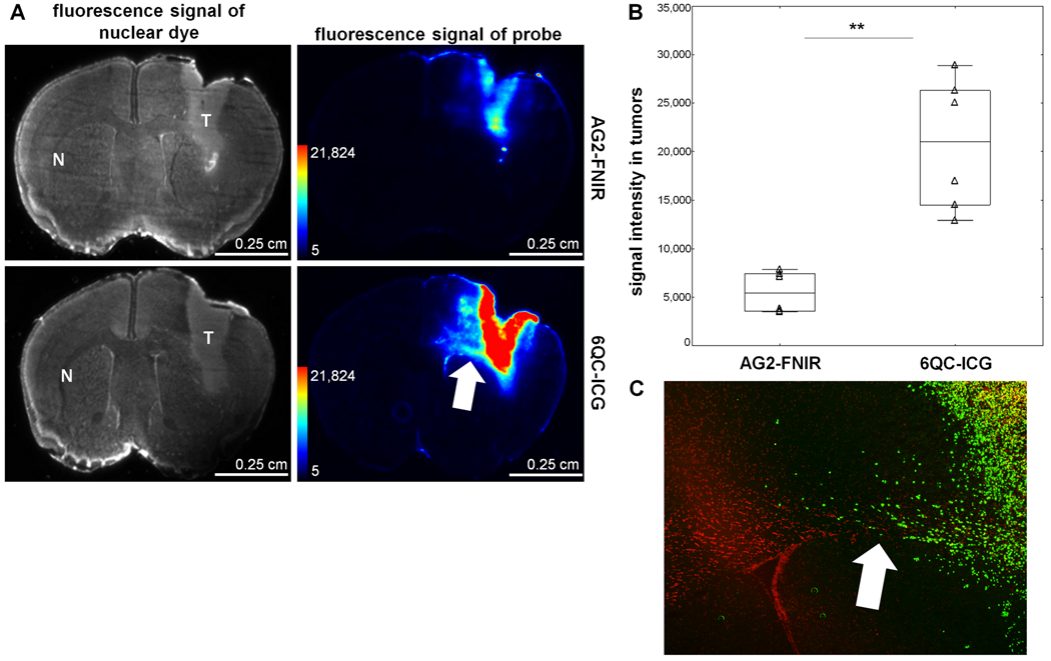
6QC-ICG shows a higher fluorescence signal intensity in the tumor than AG2-FNIR in orthotopic GBM models. **A:** Fluorescence signals of the nuclear dye showing the location of U251 tumors in brain cryosections (*first column*) and the probes in the corresponding brain cryosections (*second column*). **B:** Quantification of intratumoral fluorescence signal intensity. The *horizontal lines* represent the median, the *boxes* the 25th to 75th percentile, the *whiskers* the nonoutlier range, and the *triangles* the raw data. **p = 0.005, Mann-Whitney U-test. **C:** Microscopic image of a corresponding tissue section visualizing infiltrating human glioma cells (U251) stained with an anti–human nuclei antibody (*green*). Nuclear counterstaining is shown in *red*. Tumor-bearing mice were euthanized 24 hours after intravenous administration of 20 nmol of the probes (n = 6 sections from 2 mice for each probe). Brains were extracted, and several 10-µm cryosections were prepared and imaged simultaneously under the same conditions at ex/em 755–777/813–860 nm. Nuclei were stained with TO-PRO-3 (1 µM) and imaged at ex/em 625–650/675–725 nm. The *arrows* show tumor cells infiltrating the corpus callosum.

To avoid any possible influence of variations in tumor size, shape, and location, TNR was evaluated in coronal sections. We prepared cryosections of individual brains, measured the fluorescence, and subsequently confirmed the location of tumors by staining the nuclei in the corresponding sections. Variability among sections in individual animals was low (coefficient of variation < 15%) and TNR was higher in both NIR probes (AG2-FNIR and 6QC-ICG) than in probes containing Cy5 (Fig. 2). Although AG2-FNIR and 6QC-ICG exhibited comparable TNR values, 6QC-ICG demonstrated stronger fluorescence (Fig. 3). This may be because of its smaller molecular weight facilitating penetration into the tumor, as well as a shorter emission wavelength of the FNIR fluorophore,^22^ whose fluorescence may have been partially filtered out by our imaging system.

**FIG. 4.**
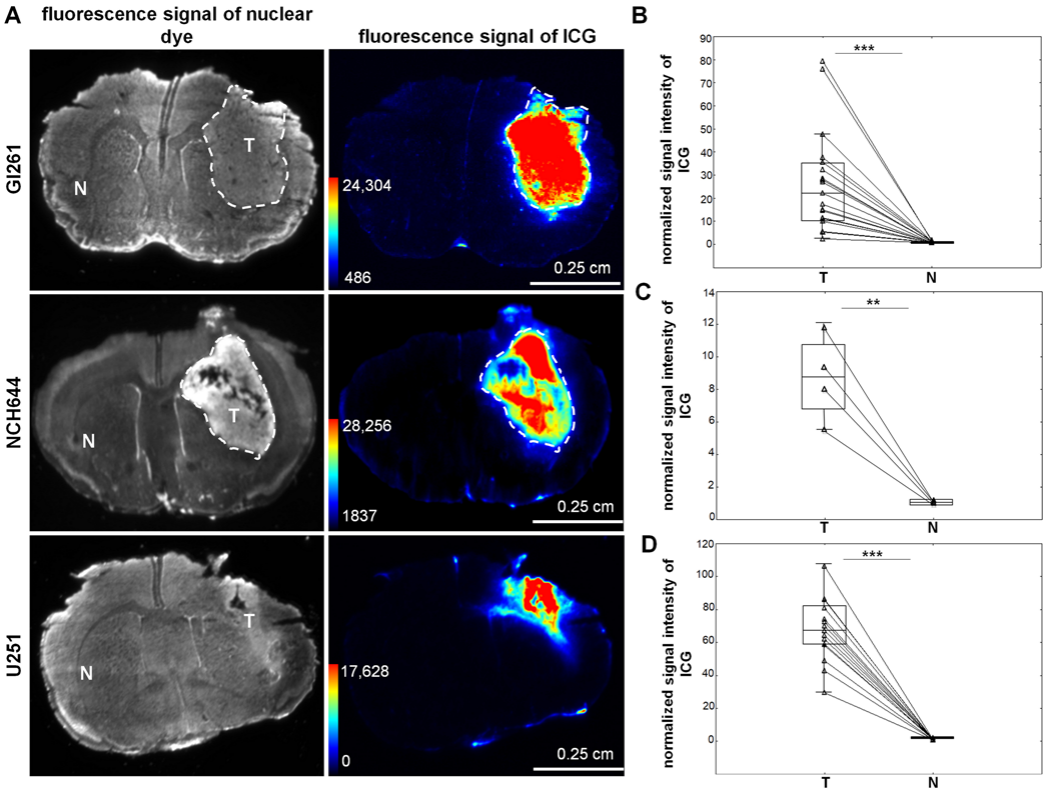
Activation of 6QC-ICG in Gl261 syngeneic, U251 xenotransplant, and NCH644 glioma stem-like cell mouse GBM models. **A:** Fluorescence signals of the nuclear dye, showing the location of the tumors in brain cryosections in Gl261, NCH644, and U251, respectively (*first column*), and the activated 6QC-ICG probe in brain cryosections (*second column*). **B–D:** Quantification of fluorescence signal in tumor and normal brain tissue in the contralateral hemisphere measured in Gl261 (B), NCH644 (C), and U251 (D) models. The fluorescence signal was normalized using the average fluorescence value in normal brain tissue in the contralateral hemisphere. The *horizontal lines* represent the median, the *boxes* the 25th to 75th percentile, the *whiskers* the nonoutlier range, and the *triangles* the raw data. ***p < 0.001, Wilcoxon signed-rank test, n = 19 and 15 mice from three independent experiments for the Gl261 and U251 models, respectively. **p < 0.01, paired t-test, n = 4 mice for NCH644 from one experiment. The *white dashed line* delineates the tumor. Nuclei were stained with TO-PRO-3 (1 µM) and imaged at ex/em 625–650/675–725 nm.

Based on these results, we chose 6QC-ICG for a detailed evaluation. In preliminary experiments, doses of 10–30 nmol per animal were tested with a better TNR after administration of the highest dose (data not shown), and a high TNR preserved for up to 48 hours (Supplementary Fig. 1). Data from previous studies^24^ indicate that, in mouse models, tumor fluorescence signal peaks 6 hours after 6QC-ICG administration; therefore, we used the 6-hour interval in all subsequent experiments. In agreement with the initial findings, fluorescence was readily detectable in both syngeneic and xenograft tumors, while signal in the normal brain remained low (Fig. 4). Tumor margins identified based on high cellular density in the Gl261 and NCH644 tumors overlapped with those seen with 6QC-ICG (Fig. 4A).

Tumors originating from the U251 cells were smaller and more invasive, as evidenced by individual cancer cells infiltrating the surrounding brain tissue and corpus callosum (Figs. 3A and 4A). In these tumors, fluorescence signal originating from the cleaved 6QC-ICG could also be detected in regions containing infiltrating cancer cells (Fig. 3C).

### 6QC-ICG Is Activated in Ex Vivo Human GBM Tissue

**FIG. 5.**
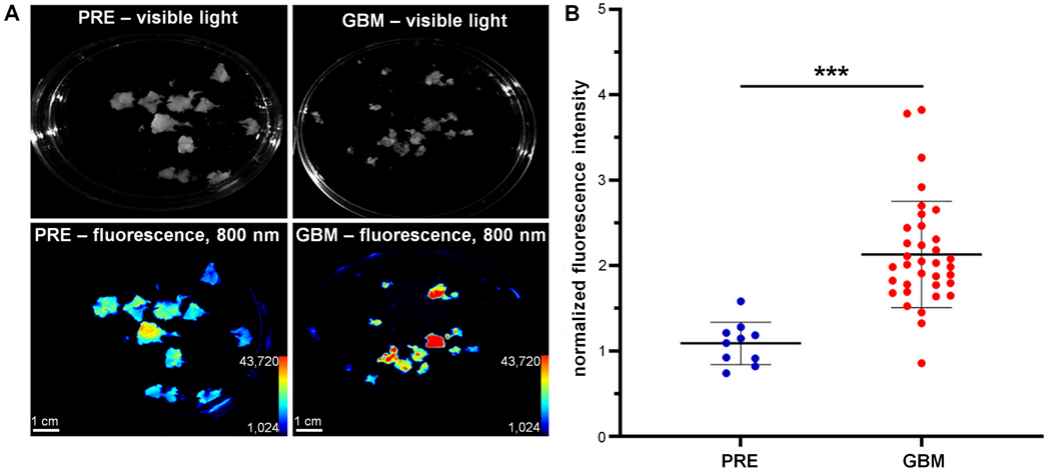
There was higher activation of 6QC-ICG in human GBM tissue than in ex vivo nonmalignant human brain tissue. **A:** Visible light (*upper row*) and fluorescence (*lower row*; ex/em 755–777/813–860 nm) images of GBM and PRE tissue. The tissue was cut into small fragments, which were incubated with 1 µM 6QC-ICG for 120 minutes at 37°C. **B:** Quantification of fluorescence signal in nonmalignant brain tissue (PRE) and GBM tissue ex vivo. Data are presented as mean ± SD in 3 different GBM donors and 1 PRE patient; identical imaging settings were used and fluorescence in individual experiments was normalized to the simultaneously processed normal mouse brain tissue. The *horizontal lines* represent the mean, the *error bars* the SD, and the *dots* the raw data. ***p < 0.001, Mann-Whitney U-test.

To assess the potential of 6QC-ICG to be specifically activated in human GBM, we performed an ex vivo experiment using tissues from patients with GBM (n = 3) and PRE (n = 1). We incubated the tissue samples with 6QC-ICG and measured probe activation at four time points (2, 5, 10, and 120 minutes). The strongest signal was observed after 120 minutes of exposure, with a statistically significant difference in probe activation between the GBM and PRE tissues (mean 1.91-fold difference, p < 0.0001) (Fig. 5). These data suggest a pronounced activation of 6QC-ICG within GBM and support its usefulness for clinical practice.

### 6QC-ICG Exhibits a Higher TNR Than 5-ALA and Can Be Visualized on Clinical-Grade Platforms

**FIG. 6.**
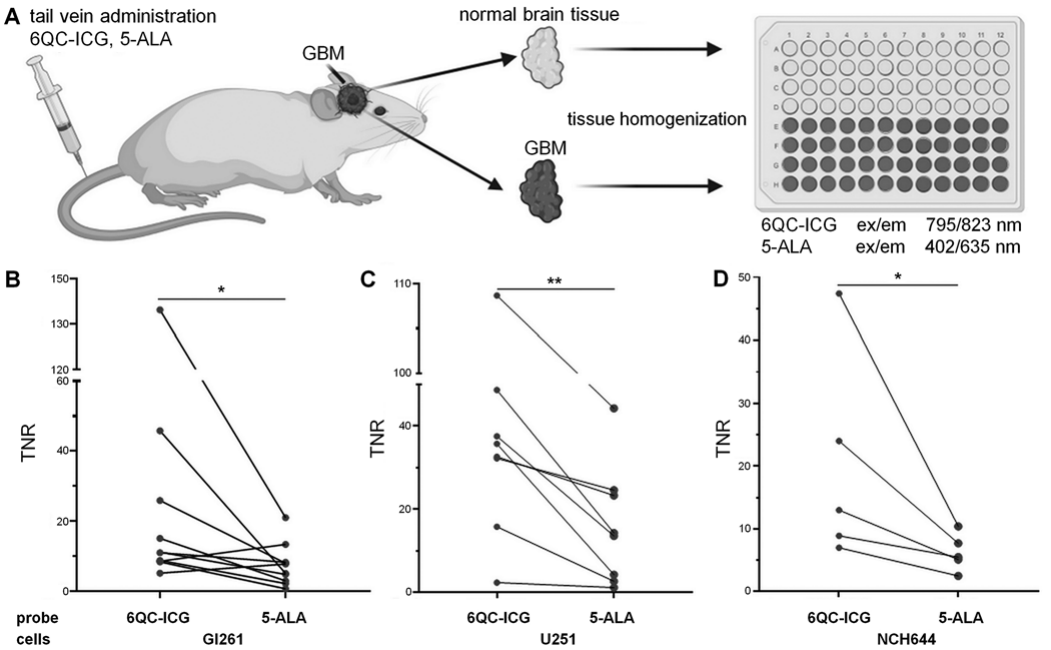
A higher TNR for 6QC-ICG than for 5-ALA was found in tissue homogenates from mouse GBM models. **A:** A schematic workflow of the spectrofluorimetric analysis of tumor and nontumorous brain tissue in mice coadministered with 6QC-ICG and 5-ALA (ex/em 795/823 nm for 6QC-ICG, ex/em 402/635 nm for 5-ALA). **B–D:** TNR of 6QC-ICG or 5-ALA in Gl261 (n = 10 mice from two independent experiments) (B), U251 (n = 9 mice from two independent experiments) (C), and NCH644 (n = 5 mice from one experiment) (D) GBM models. *p < 0.05, **p < 0.01, Wilcoxon signed-rank test. Panel A was created using BioRender.com.

The current gold standard for intraoperative visualization of GBM tissue is 5-ALA. Using experimental GBMs, we compared the abilities of 6QC-ICG and 5-ALA to distinguish between the tumor and normal brain tissue. At 6 and 5 hours^29^ before euthanasia, respectively, 6QC-ICG (30 nmol) and 5-ALA (100 mg/kg^29^) were sequentially injected via the tail vein into tumor-bearing mice. To quantitatively evaluate the fluorescence at optimal excitation/emission wavelengths, we determined the TNR in tissue homogenates from the tumor and normal brain tissue from the contralateral hemisphere using spectrofluorimetry (Fig. 6A). This assessment using tissue homogenates confirmed the results of image analysis in brain sections and revealed a significantly higher TNR for 6QC-ICG in the Gl261 (mean ± SD for 6QC-ICG 30.1 ± 16.5 vs PPIX 8.2 ± 5.7, p < 0.05) (Fig. 6B) and U251 (mean ± SD for 6QC-ICG 39.2 ± 29.4 vs PPIX 16 ± 13.5, p < 0.01) GBM models (Fig. 6C). To verify these results, the experiment was repeated using glioma stem-like cells NCH644. Consistent with previous results, TNR for 6QC-ICG (mean ± SD 20.1 ± 16.6) was significantly higher (p < 0.05) than for 5-ALA (mean ± SD 6.2 ± 3.0) (Fig. 6D).

**FIG. 7.**
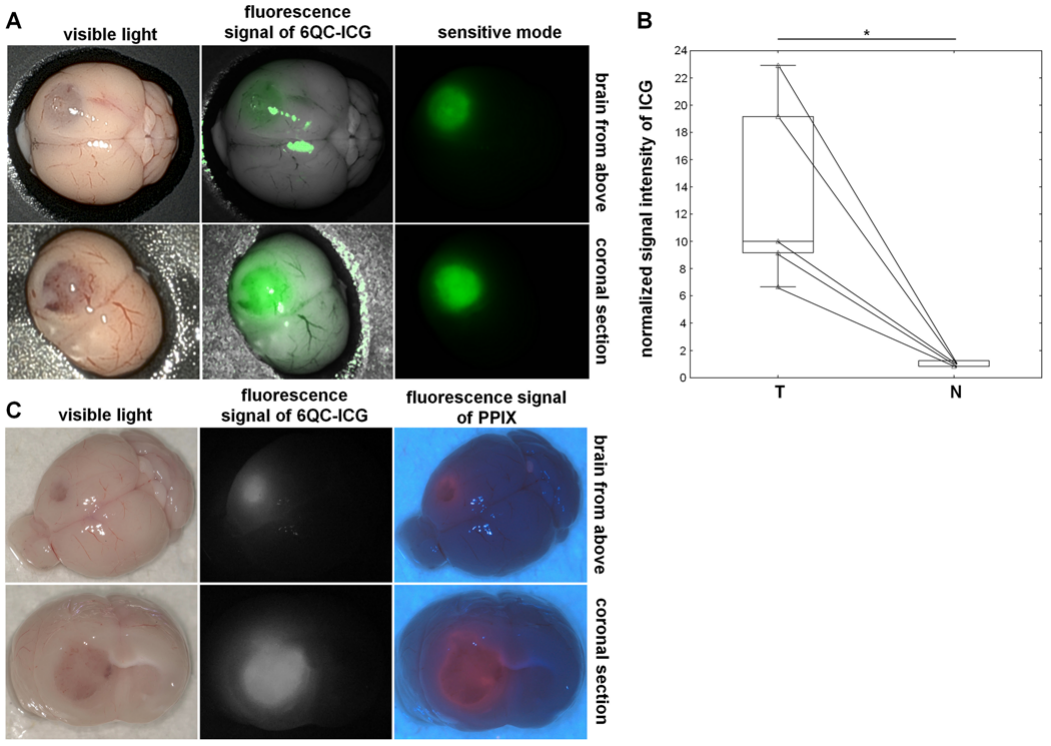
Visualization of experimental GBM with 6QC-ICG using clinical-grade imaging systems. **A:** Representative images obtained using the DaVinci Xi robotic system: images of the brain with a Gl261 tumor (whole brain and coronal section) in visible light (*first column*), corresponding NIR images (maximum ex/em ~800 nm/600–900 nm) overlaid on visible light images (*second column*), and corresponding NIR images in the sensitive mode (*third column*). **B:** Quantification of the fluorescence signal intensity. The fluorescence signal was normalized using the average fluorescence value in normal brain tissue in the contralateral hemisphere. Tumor-bearing mice (n = 5 mice with Gl261 tumors) were euthanized 6 hours after intravenous administration of 6QC-ICG 30 nmol, and the brains were extracted and stored on ice prior to imaging. The *horizontal lines* represent the median, the *boxes* the 25th to 75th percentile, the *whiskers* the nonoutlier range, and the *triangles* the raw data. *p = 0.043, Wilcoxon signed-rank test. **C:** Images obtained using the ORBEYE exoscope–representative images of the brain with a Gl261 tumor (whole brain and coronal section) in visible light (*first column*), corresponding NIR images (ex/em 730–740 nm/>800 nm) (*second column*), and corresponding blue-light images (ex/em 410–420/>450 nm) (*third column*). Tumor-bearing mice (n = 3 mice with Gl261 tumors, n = 4 mice with U251 tumors) were euthanized 6 and 5 hours after a sequential intravenous administration of 6QC-ICG 30 nmol and 5-ALA 100 mg/kg, respectively, and the brains were extracted and stored on ice prior to imaging.

We used the DaVinci Xi system to assess whether 6QC-ICG activation can be detected on a clinical-grade platform. NIR fluorescence, exhibiting a high TNR (mean ± SD 12.9 ± 4.0), was clearly detectable in regions corresponding to the tumor based on macroscopic appearance in mice administered 6QC-ICG (Fig. 7). We further compared 6QC-ICG with 5-ALA using the ORBEYE exoscope. Using an NIR mode for ICG and a dedicated blue-light mode for PPIX, we visualized coronal brain sections from tumor-bearing mice sequentially coinjected with 6QC-ICG and 5-ALA. NIR fluorescence was detectable in regions corresponding to the tumor tissue (based on macroscopic appearance and PPIX fluorescence) (Fig. 7). Despite high-sensitivity settings, the NIR signal was relatively weak and the TNR was substantially lower (mean ± SD 3.6 ± 0.9) compared with values from tissue homogenates analyzed using spectrofluorimetry and tissue sections analyzed on preclinical imaging platforms or the DaVinci Xi system. This is most likely because the NIR mode is designed for dynamic imaging of high concentrations of ICG during videoangiography and uses suboptimal excitation wavelengths (730–740 nm) for ICG. Despite these clear technical limitations in visualizing low concentrations of ICG, TNR values for 6QC-ICG were comparable to those for 5-ALA (mean ± SD 4.3 ± 1.6, p = 0.6121).

## Discussion

The differentiation between pathological and normal tissue is crucial in GBM surgery. We show that the recently developed molecularly targeted protease-activated probe 6QC-ICG may be useful for in situ visualization of GBM. All fluorescently quenched probes that we tested were activated in in vivo GBM models, and the fluorescence signal was reliably detectable for at least 24 hours after intravenous administration. 6QC-ICG exhibited the highest fluorescence signal and favorable TNR, which allowed identification of the tumor for up to 48 hours after administration. We demonstrated its activation in syngeneic and xenotransplant models, including tumors originating from GBM stem-like cells. In vitro, 6QC-ICG was activated in glioma cells and M2 macrophages, suggesting that both cell types contribute to its activation in the tumor microenvironment in vivo. The more pronounced activation of 6QC-ICG in human GBM tissue than in human nontumorous brain tissue ex vivo indicates a potential for further translation into clinical applications. Finally, our results demonstrate that the probe can be visualized on clinical-grade platforms and exhibits a higher TNR than 5-ALA, suggesting its possible applicability in FGS in GBM.

FGS offers real-time intraoperative visualization of the pathological tissue, which together with other methods, such as MRI-guided neuronavigation,^7^ iMRI,^8^ and iUSG,^9^ helps the neurosurgeon achieve maximum safe resection. 5-ALA has been used in neurosurgical practice for many years because of its utility in guiding GBM resection and improving overall survival.^10^ However, the specificity of 5-ALA for high-grade glioma (29.4%) is low according to a recent prospective multicenter study.^11^ Other studies have used nontargeted ICG, which accumulates in the tumor most likely because of the enhanced permeability and retention (EPR) effect. While nontargeted ICG identified contrast-enhancing GBM tissue with high sensitivity (98%), it had a relatively low specificity (45%).^17^

To address various limitations of the existing contrast agents, a wide number of experimental substances have been tested in GBMs, often using fluorophores that emit at longer wavelengths. The advantages of these fluorophores include decreased scattering of the emitted light and minimal tissue autofluorescence,^33^ allowing visualization of tumor that may be hidden by blood. Panitumumab (an anti-EGFR antibody) labeled with IRDye800CW had a higher TNR than 5-ALA in a mouse model.^19^ Another study used an anti-EGFR antibody cetuximab-IRDye800CW in orthotopic GBM mouse models. Compared with visible light, the use of cetuximab-IRDye800CW enabled a more extensive resection of tumor tissue.^20^ A recent early phase 1 clinical trial (NCT02901925, ClinicalTtrials.gov) tested the ABY-029 affibody targeting EGFR, but the results have not yet been published. EGFR is often overexpressed in GBMs, but with substantial intra- and intertumoral heterogeneity.^34^

Other probes for FGS in GBM target extracellular proteases. In mouse models, fluorescently labeled chlorotoxin visualized GBM, possibly by binding matrix metalloprotease 2.^35^ An NIR probe cleavable by matrix metalloproteases (MMP-750) delineated tumor margins in mouse GBM models.^36^ A possible limitation of probes that exploit the enzymatic activity of extracellular proteases may be the diffusion of the fluorophore into the surrounding tissue. The advantage of the probes tested in our study is their latent lysosomotropic effect, promoting intracellular accumulation and minimizing fluorophore diffusion.^31^

A previous study demonstrated that double-substrate (AND-gate) probes can provide increased contrast because of a decreased activation in healthy tissues.^22^ In GBM models, we observed similar TNRs for the double- and single-substrate NIR probes, with a brighter fluorescence signal in the latter. Several factors may contribute to this. An intact blood-brain barrier probably reduces permeation of the tested compounds into nontumorous brain. Consistent with this, and in line with previous studies using ICG,^17,18^ we observed very low fluorescence in the normal brain, which diminishes the main advantage (lower nonspecific activation) of double-substrate probes. In tumors, the blood-brain barrier is only partially breached,^37^ which is why the higher molecular weight of double-substrate probes may limit their passage into the tumor more than is the case in single-substrate probes. Additionally, the fluorophore used in double-substrate probes has slightly lower excitation/emission wavelengths,^22^ resulting in a less sensitive detection by our imaging system. Finally, the double-substrate probes used in this study require cleavage by cathepsins and caspase 3, but the number of apoptotic cells in the Gl261 and U251 glioma models was rather low (data not shown).

The ability of 6QC-ICG to visualize human GBM ex vivo supports the results in animal models. We observed a significantly higher fluorescence in GBM than in nontumorous brain, although the blood-brain barrier could not limit the penetration of the probe into the nontumorous brain. An even higher TNR may therefore be expected in GBM patients after intravenous administration. The approach used in our ex vivo experiments (incubation in a solution containing 6QC-ICG) might indicate a potential for topical application of 6QC-ICG into the surgical cavity, as has been proposed for some other activity-based probes.^38^ Nevertheless, our results also show that 6QC-ICG requires prolonged incubation to obtain sufficient signal, which makes it less suitable for topical application.

The TNR comparison between 6QC-ICG and 5-ALA was performed using spectrofluorimetric analysis of tissue homogenates. This approach offers the advantage of evaluating the TNR for both compounds in identical tumor and nontumorous tissue using optimal excitation/emission wavelengths. TNR values for 6QC-ICG were comparable to those obtained on preclinical imaging platforms. For 5-ALA, TNR values were similar to those reported in previous studies^29,39^ and lower than those of 6QC-ICG in all three GBM models.

We used two clinical-grade systems to visualize 6QC-ICG activation. The highly sensitive DaVinci Xi system clearly visualized experimental tumors with high contrast. The ORBEYE exoscope, which is routinely used in neurosurgical practice, visualized 6QC-ICG and also allowed simultaneous imaging using 5-ALA. However, the instrument’s sensitivity for ICG is rather low, as reported previously.^40^ Despite this, and notwithstanding the fact that the approach to convert the RBG images of PPIX fluorescence to grayscale maximized the TNR, favoring 5-ALA, we obtained similar TNRs for 6QC-ICG and 5-ALA. These results suggest that 6QC-ICG can provide a contrast superior to that of 5-ALA when high-sensitivity platforms optimized for NIR fluorophores are used.

A general constraint in using NIR probes is the availability of clinical platforms capable of superimposing fluorescence onto a visible light image with suitable sensitivity for NIR. The inherent limitations of our study relate to its proof-of-concept nature—the use of mouse models generated from GBM cells propagated in vitro. Determination of optimal dosing, persistence of adequate fluorescence signal specifically in the tumor, and establishing the optimal timing of the surgery necessitate further clinical studies. Another limitation is that direct comparison with 5-ALA using image analysis proved problematic because of the low sensitivity of the ORBEYE exoscope. Consequently, our quantitative data are mainly based on spectrofluorimetric analysis of tissue homogenates, which may be affected by the homogenization process. Future studies also need to evaluate the specificity and sensitivity of 6QC-ICG using histological analysis of the resected fluorescing tissue. This would help to assess its performance at the tumor margins and evaluate possible nonspecific activation in regions rich in inflammatory cells containing cathepsins. At the doses used, we observed no toxic effects in vivo, and the compounds were not toxic for cells in vitro. Up to 100 nmol of 6QC-ICG (9.2 mg/kg) has been previously administered in mice with experimental breast tumors with no adverse events,^23^ and several other studies have used 6QC-ICG or similar probes in mice and pigs without toxic effects^24,31,41^ (unpublished data). Nevertheless, detailed toxicology studies have not been published.

The practical application of FGS is limited by the subjective assessment of the fluorescence signal, leading to high interobserver variability. Given that the radicality of resection depends on a high contrast between the tumor and nontumorous tissue, further development of both new imaging agents and new technologies for their detection is important. Quantitative approaches that would account for the fluorescence properties of the tissue and endogenous autofluorescence may represent a further improvement.^42^

## Conclusions

Using preclinical GBM models and ex vivo human GBM tissue, we showed that the protease-activated probe 6QC-ICG is activated by glioma cells and tumor-associated macrophages, providing a high contrast between tumor and nontumorous tissue. Our data further demonstrate that, with the use of optimized and sensitive imaging platforms, protease-activated probes with ICG may offer superior contrast compared with the current gold standard, 5-ALA. These proof-of-concept findings justify further studies and suggest the potential of 6QC-ICG to become a targeted agent for intraoperative fluorescence detection of GBM.

## Acknowledgments

This research was funded by the project "Center of Tumor Ecology" (CZ.02.1.01/0.0/0.0/16_019/0000785), Operational Program Research, Development, and Education of the Ministry of Education, Youth and Sports (Czech Republic) and by the National Institute for Cancer Research (Programme EXCELES, LX22NPO5102), funded by the European Union—Next Generation EU. The research was also supported by the Ministry of Health of the Czech Republic, grant no. NV19 03 00501; the Cooperatio Program, research area "Oncology and Haematology," grant no. LM2023053 of EATRIS-CZ by the Ministry of Education, Youth and Sports (Czech Republic); and the National Institutes of Health, grant no. R01 EB028628 to M.B.

We wish to express our gratitude to Kvetoslava Vlasicova, Karin Roubickova, and Michal Ondica, Bc, for their excellent technical assistance, and Associate Professor Miroslav Zalesky for providing technical facilities.

## Disclosures

Dr. Widen reported a patent for US63495041 pending.

## Author Contributions

Conception and design: Bušek, Konečná, Widen, Bogyo, Šedo. Acquisition of data: Bušek, Konečná, Výmola, Ternerová, Výmolová, Garcia-Borja, Mateu, Pankrác. Analysis and interpretation of data: all authors. Drafting the article: Konečná, Výmola. Critically revising the article: Bušek, Konečná, Widen, Netuka, Šroubek, Bogyo, Šedo. Reviewed submitted version of manuscript: Bušek, Konečná, Garcia-Borja, Widen, Netuka, Šedo. Approved the final version of the manuscript on behalf of all authors: Bušek. Statistical analysis: Bušek, Konečná. Administrative/technical/material support: Pankrác. Study supervision: Bušek, Šedo.

## Supplemental Information

Online-Only Content Supplemental material is available with the online version of the article.*Supplementary Methods and Figure.* https://thejns.org/doi/suppl/10.3171/2024.1.JNS231137.

## Previous Presentations

Part of the results were presented as a poster at EANO 2023, Rotterdam, The Netherlands, September 21–24, 2023. The abstract (P15.03.B) was published in *Neuro-Oncology*, Volume 25, Issue Supplement 2, September 2023, pages ii109–ii110.
