# Supplementary material for: Molecularly targeted protease-activated probes for visualization of glioblastoma: a comparison with 5-ALA
Source: J Neurosurg. 2024 Mar 29:2024.1.JNS231137. doi: 10.3171/2024.1.JNS231137 (PMC11010726; doi:10.3171/2024.1.JNS231137)
Supplement: Online-Only Content — Supplemental material is available with the online version of the article.Supplementary Methods and Figure. https://thejns.org/doi/suppl/10.3171/2024.1.JNS231137. [file SupplementaryMethodsandFigure_JNS23-1137.pdf]

ONLINE ONLY

Supplemental material

Molecularly targeted protease-activated probes for visualization of glioblastoma: a comparison with 5-ALA

Konečná et al.

https://thejns.org/doi/abs/10.3171/2024.1.JNS231137

DISCLAIMER The *Journal of Neurosurgery* acknowledges that the following section is published verbatim as submitted by the authors and did not go through either the *Journal's* peer-review or editing process.

© 2024 The authors, CC BY 4.0 (http://creativecommons.org/licenses/by/4.0/)

Supplementary Methods

Tissue samples: Biopsy material was obtained from consenting patients with newly diagnosed glioblastoma or pharmacoresistant epilepsy (PRE) at the Department of Neurosurgery and Neurooncology, Military University Hospital, Prague. The study was approved by the Institutional Ethics Committee (study approval number 108-39/4-2014-UVN) and it was in accordance with the Declaration of Helsinki. The diagnosis of glioblastoma was established according to the current WHO classification [1].

Cell culture: Cells were cultured under standard conditions at 37°C in a humidified atmosphere of 5% CO_2 in air. The human glioma cell line U251 was obtained from ATCC (LGC Standards, UK) and was cultured in Dulbecco's Modified Eagle's Medium (DMEM, Sigma Aldrich, Czech Republic) supplemented with 10% fetal calf serum (FCS, Sigma Aldrich, Czech Republic). The murine glioma cell line Gl261 was obtained from the Division of Cancer Treatment and Diagnosis Tumor repository, National Cancer Institute, USA and was cultured in RPMI-1640 (Sigma Aldrich, Czech Republic) supplemented with 10% FCS.

Glioma stem-like cells were derived from resected glioblastoma tissues as previously described[2]. Tissue was dissociated using a Papain Dissociation System following the manufacturer's protocol (Worthington Biochemical Corporation, USA). The resulting single cell suspension was cultivated in DMEM/F-12 (Sigma Aldrich, Czech Republic), GlutaMAX™ (1%, Gibco, Thermo Fisher Scientific, USA), B-27™ Supplement minus vitamin A (2%, Gibco, Thermo Fisher Scientific, USA), 100 U/mL penicillin, 100 µg/mL streptomycin (Sigma-Aldrich, Czech Republic), 20 ng/mL EGF and 20 ng/mL FGF (Peprotech) in non-adherent cell culture flasks. The glioma stem-like cell line NCH644 was obtained from Cell Lines Service (Germany) and was cultured under the same conditions.

Macrophages were differentiated from monocytes obtained from the buffy coats of healthy donors. Buffy coats (n=2) were collected at the Institute of hematology and blood transfusion, Prague (ethical committee approval reference number 06/11/2020); informed consent was obtained from all subjects. Peripheral blood mononuclear cells (PBMCs) were isolated by density gradient centrifugation. The buffy coat sample was layered onto Ficoll Paque (Cytiva) and centrifuged (1800 rpm, 40 minutes, room temperature). The PBMC fraction was collected using a pasteur pipette, washed twice with PBS and subsequently red blood cells were lysed (RBC lysis solution, Miltenyi). Monocytes were isolated from PBMC using the pan-monocyte isolation kit (Miltenyi). The purity of isolated monocytes, determined as the percentage of CD14+ cells by flow cytometry (CD14-FITC, Sony), was more than 90%. Monocyte-derived M2-polarized macrophages were obtained after seven days of cultivation in RPMI-1640 supplemented with 10% heat-inactivated FCS and 50 ng/ml M-CSF (Miltenyi). Media were replaced on day four. IL-4 (20 ng/ml; Miltenyi) was added 24 hours before cell harvesting. The M2-like phenotype was validated by the expression of CD163 and CD206 and the absence of CD80 (CD163-PE; CD206-APC; CD80-APC; Sony).

Animals and ethics statement: All animal experiments were performed according to protocols approved by the institutional commission and the Ministry of Education, Youth, and Sports of the Czech Republic (MSMT-270/2020-2). Surgical procedures were performed under intramuscular anesthesia (Ketamine 100 mg/kg and Xylazine 10 mg/kg) with efforts to minimize animal suffering. Immunodeficient (NOD.129S7(B6)-Rag1tm1Mom/J) and immunocompetent (C57BL/6J) 6–10 week-old male mice were chosen for the implantation of human and mouse glioma cells, respectively.

Orthotopic glioblastoma model: Experimental tumors were generated by stereotactic injection of glioma cells (Gl261, U251, NCH644) into mouse brains as previously described[3]. Animals

were anesthetized and placed in a stereotactic frame (Stoelting), in which coordinates were set with a 0.1 mm accuracy (3 mm caudal to bregma, 2.5 mm lateral to the sagittal suture). Cell suspension in serum-free medium (5 µl, 500 000 cells for U251 and NCH644, 100 000 for Gl261 cells) was injected at a speed of 1 µl/minute with a Hamilton syringe 3 mm deep into the right hemisphere. Mice were closely followed and experiments with fluorescent agents were performed when symptoms appeared.

Fluorescent agent preparation and administration: For *in vivo* experiments, protease-activated probes (6QC-ICG, 6QC-Cy5, AG2-FNIR, AG2-Cy5; stock 10 mM in DMSO[4] were diluted in a solution containing 10% DMSO (Sigma-Aldrich D2650), 30% polyethylenglycol (PEG400, 91893 Sigma-Aldrich) and 60% PBS and administered intravenously via tail vein (10 to 30 nmol per mouse in 100 µl). Each probe was evaluated separately in tumor-bearing mice. 5-aminolevulinic acid (5-ALA, Gliolan 30 mg/ml 1x1.5g, Photonamic GmbH) was dissolved in sterile saline at a concentration of 30mg/ml and administered intravenously via the tail vein (128.2 mg/kg, corresponding to 100 mg/kg of 5-ALA[5]). For experiments comparing 6QC-ICG with 5-ALA, both substances were sequentially co-administered to tumor-bearing animals 6 hours and 5 hours, respectively, prior to euthanization.

Fluorescence imaging on preclinical and clinical platforms: The Xtreme imaging system (The Center for Advanced Preclinical Imaging, First Faculty of Medicine Charles University, ex/em 630/700 nm for Cy5; ex/em 730/790 nm for ICG and FNIR) was used for the evaluation of protease-activated probes 2 and 24 hours after probe administration. Subsequent analyses of 6QC-ICG were performed on the ChemiDoc MP imaging system (ex/em 755–777/813–860 nm). Tumor-bearing mice were sacrificed 6 hours after probe administration and then brain cryosections were prepared for visualization of fluorescent signal.
To visualize 6QC-ICG on clinical grade platform, we used coronal section of tumor-bearing brains. The DaVinci Xi robotic system (Intuitive Surgical, ex/em 736 nm/600-900 nm, Department of Urology, Military University Hospital) and the near-infra red mode (NIR, ex/em 730–740 nm/>800 nm) on the Orbeye exoscope (Olympus, Department of Neurosurgery and Neurooncology, Military University Hospital) were used. 5-ALA was imaged using a blue mode (ex/em 410–420/>450 nm) of the Orbeye exoscope (Department of Neurosurgery and Neurooncology, Military University Hospital).
Quantification in frozen sections was performed using 10 µm and 50 µm thick coronal plane cryosections (prepared on the Cryotome Leica CM 1950, Leica, USA). The 50 µm cryosections were visualized on the ChemiDoc MP imaging system (ex/em 755–777/813–860 nm) and analyzed. To validate the location of the tumor, the same cryosections were subsequently stained with a nuclear dye (To-Pro3 Iodide, Thermofisher Scientific; 1 µM solution in PBS, 15 minutes, RT) and visualized on the ChemiDoc MP imaging system (ex/em 625–650/675–725 nm). To evaluate the signal at the tumor margins, a mask highlighting the tumor was generated in nuclear dye-stained images and transferred into the corresponding position in the fluorescence images with the ICG signal.

Quantitative image analysis: Image-J (National Institutes of Health, Bethesda, MD, US) was used to quantify the fluorescence signal and TNR was calculated to evaluate the ability of individual probes to visualize tumor tissue. The input for TNR calculations were images of coronal plane brain sections or cryosections with tumor obtained on preclinical and clinical visualization platforms. The tumor (T) and the area of a corresponding size in the contralateral hemisphere (normal brain tissue, N) were selected as regions of interest (ROI). The median fluorescent intensity of the ROI's was used to calculate the TNR for each section. To compare 6QC-ICG and 5-ALA, images captured from identical position in visible light, NIR, and blue light mode of the Orbeye digital video microscope were used. Tumor and normal brain tissue

in the contralateral hemisphere were selected as ROIs in visible light images and then projected onto the NIR and blue light images. For 6QC-ICG, the TNR was calculated as described above. 5-ALA RGB images were first converted to a greyscale image. This was done by calculating the scalar product of the pixel colors and a projection RGB vector. The projection vector was defined as a vector which most separated colors in a tumor region from colors in a healthy brain tissue region. For each sample, a single projection vector was computed by solving an optimization problem that maximized the distance between median colors of both regions. The mean projection vector was then used to perform the grayscale conversion of all RGB images. The resulting grayscale images were then analyzed as in the case of 6QC-ICG images.

Immunohistochemistry: Immunofluorescence staining was performed in 10 μm cryosections as previously described[6]. The sections were fixed with 4% paraformaldehyde (10 min at RT), permeabilized (0.1% Triton X-100, 5 min at RT), and blocked with 10% FCS and 1% bovine serum albumin in TBS, 60 min at RT). Anti-human nuclei antibody (Anti-Nuclei Antibody, clone 3E1.3, MAB4383, Sigma-Aldrich, Czech Republic; 1:500 in 1%BSA in Tris-buffered saline [TBS], ON at 4°C) followed by an anti-mouse Alexa Fluor 488 secondary antibody (Invitrogen, Thermo Fisher Scientific, US; 1:500 in 1%BSA in TBS, 1 hour at RT) were used, nuclei were counterstained using Hoechst 33258 (Merck, 50 ng/mL). Samples were mounted in Aqua Poly/Mount (Polysciences, Hirschberg, Germany), and viewed on an Olympus IX70 microscope (Olympus Czech Group, Prague, Czech Republic). Images were taken with an Orca-flash 4.0 camera (Hamamatsu, Hamamatsu, Japan).

6QC-ICG activation in human glioblastoma tissue *ex vivo*: Freshly resected glioblastoma or pharmacoresistant epilepsy tissues were transported from the operation theatre in MACS Tissue Storage Solution (Miltenyi) on ice. Tissue samples were washed with PBS and cut into pieces. Subsequently, the tissue pieces were transferred to a 50 ml falcon tube containing a preheated solution of 6QC-ICG (1 μM in DMEM F12 supplemented with 10% serum, 37°C) and kept in the dark at 37°C. After 2, 5 and 10 minutes of incubation, tissue pieces were taken from the falcon tube and immediately imaged using ChemiDoc MP imaging system (IRDye 800 NIR setup, ex/em 755–777/813–860 nm, manual exposure time 1.7s, no binning). The pieces were then transferred back into the 1 μM 6QC-ICG solution. Final imaging was performed after 120 minutes of incubation with 6QC-ICG. The mean fluorescence intensity was calculated using ImageLab software (Bio-Rad). Because simultaneous comparison for individual human tissues was not logistically feasible, we used the mean fluorescence intensity measured in healthy mouse brain tissue (NOD.129S7(B6)-Rag1tm1Mom/J, 8-12 weeks old) simultaneously processed with the human material to normalize fluorescence intensities in individual experiments.

6QC-ICG activation *in vitro*: U251 (2.5×10^5 cells per well in a 6 well plate) and Gl261 (3.5×10^5 cells per well in a 6 well plate) glioma cells were seeded in their corresponding media. After 72 hours, the media were replaced by phenol red-free media with 1 μM 6QC-ICG or vehicle (DMSO). The cells were incubated for 6 hours under standard conditions. Subsequently, cells were washed twice with PBS and harvested with Trypsin-EDTA (Sigma-Aldrich). Cells were centrifuged (348 g at RT, 5 minutes) and resuspended in 0.6 ml of PBS. The cell suspension (100 μl) was transferred in triplicates to 96-well black flat bottom plates and fluorescence was measured. PBS was used as a control. Monocyte-derived M2-polarized macrophages were incubated with 1 μM 6QC-ICG as described above. Glioma stem-like cells were cultured as described above. The spheres were transferred into Falcon tubes, centrifuged (125 g at RT, 4 minutes), and the supernatant was aspirated until 3 ml remained. 6QC-ICG (f.c. 1 μM) or vehicle (DMSO) was added, and spheres were incubated under standard conditions. After 6 hours, the spheres were centrifuged (125 g at room temperature for 4

minutes), washed 3 times with PBS, and transferred to a 96-well plate. All fluorescence measurements were performed on a microplate fluorimeter Infinite M1000 (Tecan, Grödig, Austria) at ex/em 795/823 nm.

Flow cytometry analyses: Glioma cells and macrophages were harvested after 6 hours of incubation with 1 µM 6QC-ICG or vehicle (DMSO) as described above, and fluorescence intensity was measured using BD FACSverse (640 nm laser, APC-Cy7). Data were analyzed using FLOWJO software (BD) and the mean fluorescence intensity was used for comparative analyses.

Confocal microscopy: Adherently growing cells and sphere-forming glioma stem-like cells were imaged after 2 hours of incubation with 1 µM 6QC-Cy5 or vehicle (DMSO) using a FluoView FV300 Confocal Microscope (Olympus, Shinjuku, Japan) equipped with a 633 nm HeNe laser.

Spectrofluorimetric analysis of tissue homogenates: Pieces of experimental tumors and healthy mouse brain tissue were used for spectrofluorimetric analysis. All procedures were performed on ice. Tissue samples were mechanically homogenized in 200 µl of dissociation buffer (PBS with protease inhibitors E64 [f.c. 50 µM], AEBSF [f.c. 200 µM] and pepstatin [f.c. 25 µM] to prevent *ex vivo* activation of 6QC-ICG in the tissue) using a one milliliter pipet tip and then sonicated for three minutes in a Bandelin SONOREX™ SUPER Ultrasonic bath with ice cold water. The samples were triturated again and sonicated for additional three minutes. 150 µl of the suspension was transferred to a black 96-well plate and fluorescence intensity was measured using Infinite M1000 (6QC-ICG ex/em 795/823 nm and 5-ALA ex/em 402/635 nm). The data were normalized to tissue sample weight. Normalization to total protein produced similar results.

Protein quantification: Protein concentration was analyzed using a detergent-compatible protein assay (BioRad) according to the manufacturer´s protocol. The absorbance was measured using a Tecan spectrophotometer at 750 nm.

Statistical analyses: Statistical analyses were performed with STATISTICA 14 (Tibco Software, Palo Alto, CA, USA) and GraphPad Prism 8.0.2 (GraphPad Software, San Diego, CA, USA). Paired t-test for normally distributed data or Wilcoxon matched pair test and Mann-Whitney U-test were used to verify the difference between groups. A two-sided $p < .05$ was considered statistically significant.

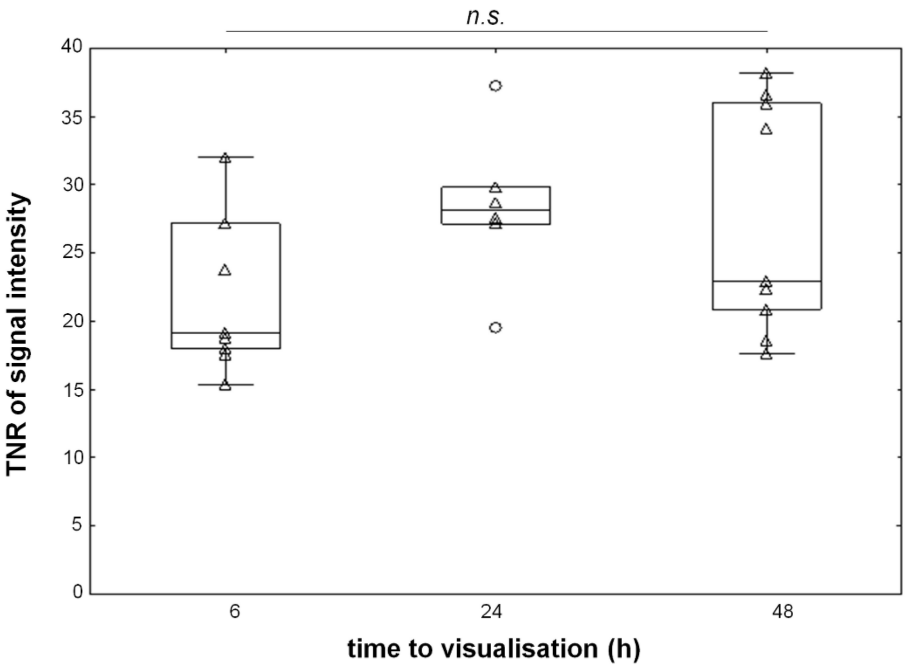

Supplementary Fig. 1. Tumor to normal ratio (TNR) for 6QC-ICG in the Gl261 model at different time points. Gl261 tumor bearing mice were euthanized 6 (n=3), 24 (n=3), and 48 hours (n=3) after intravenous administration of 30 nmol of 6QC-ICG. Brains were extracted, 50 µm cryosections (three for each mouse) were prepared and fluorescence was quantified using ChemiDoc MP at ex/em 755–777/813–860 nm. Line – median; box – 25th to 75th percentile; whiskers – non-outlier range; triangles – raw data. p= .18 for 6 vs 24hours, Kruskal-Wallis test.

Supplementary References

1. Louis DN, Perry A, Wesseling P, et al. The 2021 WHO classification of tumors of the central nervous system: a summary. *Neuro Oncol*. 2021;23(8):1231-1251.
2. Sana J, Busek P, Fadrus P, et al. Identification of microRNAs differentially expressed in glioblastoma stem-like cells and their association with patient survival. *Sci Rep*. 2018;8(1).
3. Busek P, Stremenova J, Sromova L, et al. Dipeptidyl peptidase-IV inhibits glioma cell growth independent of its enzymatic activity. *International Journal of Biochemistry and Cell Biology*. 2012;44(5):738-747.
4. Yim JJ, Tholen M, Klaassen A, Sorger J, Bogyo M. Optimization of a protease activated probe for optical surgical navigation. *Mol Pharm*. 2018;15(3):750-758.
5. Swanson KI, Clark PA, Zhang RR, et al. Fluorescent cancer-selective alkylphosphocholine analogs for intraoperative glioma detection. *Neurosurgery*. 2015;76(2):115-123.
6. Balaziova E, Vymola P, Hrabal P, et al. Fibroblast activation protein expressing mesenchymal cells promote glioblastoma angiogenesis. *Cancers (Basel)*. 2021;13(13).
